# Prioritising interventions for preventing mental health problems for children experiencing adversity: a modified nominal group technique Australian consensus study

**DOI:** 10.1186/s40359-021-00652-0

**Published:** 2021-10-24

**Authors:** Teresa Hall, Suzy Honisett, Kate Paton, Hayley Loftus, Leanne Constable, Harriet Hiscock

**Affiliations:** Centre of Research Excellence in Childhood Adversity and Mental Health, Centre for Community Child Health, Murdoch Children’s Research Institute, The Royal Children’s Hospital, 50 Flemington Rd, Parkville, VIC 3052 Australia

**Keywords:** Child mental health, Childhood adversity, Nominal group technique, Consensus study

## Abstract

**Background:**

Despite the well-established link between childhood adversity and mental health problems, there is a dearth of evidence to inform decision making about the most acceptable and feasible interventions for preventing mental health problems for children experiencing adversity. Expert consensus is an important input into evidence-informed policy and practice but is often employed at the national level which misses important local contextual factors shaping decision making. This study aimed to: (1) reach consensus on local priority interventions for preventing mental health problems for children living with adversity in Wyndham, Victoria; and (2) understand the enabling factors and barriers to implementing these interventions.

**Methods:**

This study employed six online modified nominal group technique (NGT) workshops with 19 stakeholders; intersectoral service providers from health, social and education sectors and caregivers of children aged 0–8 years.

**Results:**

Three interventions reached consensus among the mixed stakeholder groups as being a high or very high priority for implementation in Wyndham: nurse home visiting, parenting programs and community-wide programs. Key rationales were the ability for these interventions to act as a gateway for families to increase their knowledge about topics immediately relevant to them (i.e. parenting), increase their knowledge about available supports and build relationships with service providers.

**Conclusions:**

Local priorities for preventing mental health problems for children living with adversity emphasized relational approaches to service provision and were shaped by the availability of existing interventions and supports in the locality. The NGT was found to be an effective method for prioritising evidence-based practice interventions in health settings, engaging local stakeholders, and identifying enablers and barriers to implementation.

**Supplementary Information:**

The online version contains supplementary material available at 10.1186/s40359-021-00652-0.

## Background

Anxiety and depressive disorders are major sources of disease burden in children and young people in Australia and globally [1, 2]. There is a significant social gradient of mental health burden, with a much higher prevalence of mental health problems among children and young people experiencing adversity because of their socio-economic, health, geographic and/or family circumstances [3]. Within this population, a specific set of exposures known as Adverse Childhood Experiences (ACEs) has been defined as “exposure during childhood or adolescence to environmental circumstances that are likely to require significant psychological, social, or neurobiological adaptation by an average child and that represent a deviation from the expectable environment” [4]. An expanded definition of ACEs includes childhood maltreatment (e.g. physical, verbal or sexual abuse), household dysfunction (e.g. parental divorce, family substance abuse, parental mental illness, maladaptive parenting), community dysfunction (e.g. witnessing physical violence) and peer dysfunction (e.g. stealing, discrimination, bullying) and socio-economic adversity [5–7]. Collectively, these can be viewed as family adversity.

ACEs are an important target for intervention in childhood because they are major contributors to the disease burden from mental disorders across the lifespan [8]. ACEs such as exposure to family violence and parent mental illness cluster in families experiencing adversity, with around two-thirds of children experiencing or exposed to multiple ACEs [7]. Whilst child maltreatment accounts for 16–33% of depression, anxiety and self-harm in Australian adults [9], other ACEs also contribute to poor adult mental and physical health outcomes [10]. In Australia, where this study was undertaken, it is estimated that two-thirds (64%) of Australian children have experienced at least one ACE [11], with children from Aboriginal and Torres Strait Islander and ethnic minority backgrounds at a significantly increased risk of experiencing two or more ACEs [12]. The high prevalence of ACEs [13, 14], coupled with the increasing evidence of their significant contribution to most classes of mental disorders [14, 15], suggests that interventions to prevent or reduce the impact of ACEs could mitigate a substantial population burden of mental disorders [15].

Despite the well-established link between family adversity and mental health problems, there is a dearth of evidence to inform decision making about the most acceptable and feasible interventions for preventing or mitigating mental health problems for children experiencing adversity. Expert consensus is an important input into evidence-informed policy and practice that is widely employed in the mental health field [16]. Under certain conditions, expert consensus methods have strong validity by tapping into the ‘wisdom of crowds’ [16]. Specifically, a diverse range of stakeholders with imperfect expertise, who can make decisions independently and in a de-centralised manner, with a mechanism for aggregating their judgements, produce better judgements than individual experts acting alone [17].

While the Delphi consensus method is the most common method to achieve expert consensus [16], the nominal group technique (NGT) is another evidence-based consensus method. The NGT method combines interactive individual and group phases to reach consensus and as such has the benefit of generating qualitative data to garner rich accounts of perspectives on a given topic [18–20]. Both the Delphi and NGT consensus methods are vehicles that translate knowledge derived from research evidence into practice and maximise rigour through participant anonymity, iteration of ratings, controlled feedback and statistical evaluation of consensus [21].

A recent unpublished Delphi study established priority interventions by national-level experts for Australian children living with adversity [23]. This Delphi study identified seven priority interventions for ACEs: community-wide interventions; parenting programs; home-visiting programs; psychological interventions; school-based anti-bullying interventions; psychological therapies for children exposed to trauma; and the specific Positive Parenting Program (Triple P){Sanders, 2014 #466}. However, context is important for effective knowledge translation [18, 23, 24] and expert consensus reached at a national level may miss important contextual factors influencing priorities for communities at a local implementation level [24, 25].

We conducted this NGT consensus study to bridge the local evidence to practice gap by providing local expert judgment on what evidence-based interventions for preventing child mental health problems for children aged 0–8 years living with adversity are most likely to be effective in the Wyndham local government area in the state of Victoria, Australia. Hence, this study had two objectives:

1. To reach consensus on priority interventions for preventing or mitigating mental health problems for children living with adversity in Wyndham, Victoria; and
2. Understand the enabling factors and barriers to implementing these interventions from the perspectives of health, social and education sector service providers and caregivers of children aged 0–8 years.

## Methods

### Methodological approach

This study is part of a broader research project that aims to co-design, test, evaluate and scale-up an integrated Hub model of care for families living with adversity in two community health centres. This study was conducted as part of the formative research phase informing the co-design of the Hub model in Wyndham, Victoria. A local advisory group constituted of community and intersectoral service providers and a family representative oversaw the development of the study design and recruitment (including advertising materials).

This study employed six online modified NGT workshops with intersectoral service providers and caregivers of children aged 0–8 years. We modified the NGT by structuring the workshops around six types of evidence-based interventions (rather than beginning with idea generation) and delivering the workshops online. The modified NGT method involved: (1) presentation of the research topic and six types of evidence-based interventions, (2) individual rating of these interventions for their priority in Wyndham, (3) group discussion about the ratings and enabling factors and barriers to implementation of the interventions, (4) re-rating by individuals and (5) final group discussion.

The six types of evidence-based interventions for preventing child mental health problems for children aged 0–8 years living with family adversity were identified through an umbrella review {Sahle, 2021 #447} that informed a Delphi process conducted with national experts as part of the broader research project) (unpublished data, Sahle et al.). These evidence-based interventions are summarised in Table 1. We used the six types of evidence-based interventions to structure the NGT process to maximise comparability between the priorities of national-level experts elicited through the Delphi study and local priorities of Wyndham stakeholder groups. Further, we wanted to understand local barriers and enablers to evidence-based practice. Such a comparison is critical for furthering understanding of the knowledge translation process for family adversity across a range of contexts.

**Table 1:** Overview of the six evidence-based interventions

Intervention	Short description
Parenting programs	Designed to help parents and caregivers develop skills, strategies and confidence to parent their children positively. Delivered to parents of children 0–16 years in a variety of group and one on one settings. Positive Parenting Program (Triple P){Sanders, 2014 #466} was provided as an example
School-based anti-bullying programs	Delivered in schools to teach children self-awareness and relationship skills, how to respond to bullying and make responsible decisions
Psychological therapy for children exposed to trauma	Individual and group-based talk therapyprovided to parents with a mental illness and their children 0–4 years. Parental mental illness is one of the ACEs with the strongest evidence for negatively impacting child mental health {Sahle, 2021 #447}. Evidence-based therapies included cognitive behavioural therapy and interpersonal therapy
Community-wide programs	Programs that take a whole-of-system approach to build community connectedness to better support families. They utilise existing community assets and are managed by partnerships between health, education, social services, and voluntary sectors. Elements may include: outreach and home visits; support to families and parents; and support for good-quality play, learning and childcare facilities
Nurse home visiting programs	Nurses conduct home visits for parents with children aged 0–2 years. Nurse home visiting programs are part of universal services offered to all families across Australian jurisdictions but also include more intensive home visitation programs offered to at-risk families. They aim to support a positive home environment, facilitate positive parent-infant relationships, teach parents coping and problem-solving skills and link families into support services. These interventions are targeted at children in the first few years of the 0–8 years age range of interest for this study
Economic and social programs	Financial, employment and housing support provided by the government to low-income families

We used NGT methods instead of other consensus methods (i.e. a Delphi expert consensus study) to increase the accessibility for stakeholders with limited research literacy; reduce the time burden on participants and reduce the influence of response bias resulting from intergroup dynamics and the researchers’ presence [18, 19]. The NGT workshops also served as a stakeholder engagement strategy to support the co-design process of the Hub model and build effective relationships for the uptake and implementation of the Hub [27]. The use of a web-based platform to host the NGTs and other group research has been shown to have high acceptability and feasibility and increase access for hard-to-reach populations [28–30].

### Setting

The City of Wyndham is a metropolitan local government area (LGA) in the outer South-Western suburbs of Greater Melbourne and is home to 57,508 families with children [31]. More than half of Wyndham’s children aged 0–4 years have two parents born overseas and around 70% had at least one parent born outside of Australia [32]. Detecting and responding to adversity is a key concern in Wyndham due to several population risk factors for childhood adversity, including an unemployment rate higher than the Greater Melbourne average (5.8% compared to 4.8%) [33]. The Australian Early Development Census estimates that approximately one quarter of children in Wyndham are vulnerable in at least one developmental domain, compared to less than one in five children Victoria-wide [32]. Mental health and family violence are the two most common reasons for referral to the Enhanced Maternal Child Health (MCH) program in Wyndham. The need to develop responsive service models for childhood adversity in Wyndham is paramount given that the number of children experiencing adversities is likely to increase as the population of children in Wyndham more than doubles over the next 18–20 years [33]. Further, Wyndham LGA was a ‘hot spot’ for COVID-19 transmission and experienced increased family employment stress (a 102% increase compared to pre-COVID-19) [34].

### Participants

Nineteen participants from two stakeholder groups from Wyndham participated in the study: service providers from health, social, child and family welfare, community and education services (*n* = 17) and primary caregivers of children aged 0–8 years (*n* = 2). This number of participants is within the typical range for NGT studies of 8–20 participants [18]. All participants met the following inclusion criteria: adults aged 18 years or over; live in, access or provide health, social, child and family welfare, community, legal, financial or educational services in Wyndham; and provide informed consent. Caregivers provided care to at least one child aged 0–8 years.

The research team recruited participants using convenience and snowball sampling. Service providers serving on local council working groups for children and family services and vulnerable children were invited to participate in the study. Service providers were sent an invitation email containing the study flyer and asked to nominate other relevant practitioners whom the research team subsequently contacted. Caregiver participants received the flyer through local networks. The flyer linked participants with a REDCap database which displayed the relevant Participant Information Statement where they could enter their contact details. Participants were recruited from September–November 2020.

Table 2 displays the demographic information for nominal group participants. All participants were women. Service providers were practitioners from a range of intersectoral services, most of whom had been in their role for two years and provided early education services and disability inclusion services. Due to recruitment challenges related to COVID-19 restrictions, only two caregivers participated in the study.

**Table 2:** Demographics of nominal group participants

Stakeholder type	*n*	Age in years median (range)	Years in current role median (range)
*Service providers*	17	35–44 (25–34 to 55–64)	2 (1–4)
Health	3	35–44 (25–34 to 35–44)	1 (1–3)
Child and family	3	35–44 (25–34 to 35–44)	2 (2–3)
Social sector	2	35–44 (35–44 to 45–54)	2 (2)
Early education and disability inclusion	7	45–54 (25–34 to 55–64)	1.5 (1–4)
Drug and alcohol	2	35–44 (35–44)	2 (2)
Caregivers	2	25–34 (25–34 to 35–44)	–
Overall	19	35–44 (25–34 to 55–64)	2 (1–4)

Health sector practitioners include specialists and allied health professionals; early education and disability inclusion practitioners include childcare and kindergarten providers, and other services who work with children with additional or unique learning needs; social sector practitioners including services providing a wide range of social supports for adults (including parents); child and family sector practitioners provide targeted supports for families and their children, usually with a focus on parenting; drug and alcohol practitioners provide counselling and support services for people living with alcohol and other drug challenges

### Data collection

**Fig. 1:**
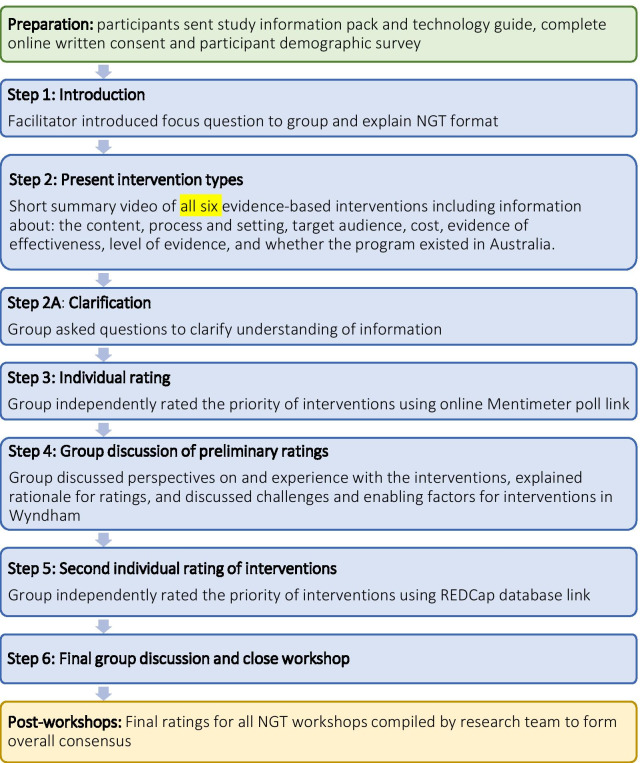
Preparation, data collection and data analyses stages of the online NGT workshops

Six NGT workshops were conducted using Zoom web conferencing platform during October and November 2020; five service provider workshops and one caregiver workshop. Figure 1 displays the preparation, data collection and analysis stages of the workshops. All stages of the workshop were conducted in one session with all participants present for the whole session. Each workshop was audio-recorded, involved 2–4 participants, and lasted on average 66.3 min (range: 61–74 min). First author (TH) facilitated the online NGT workshops using a Facilitation Guide and author (KP) moderated the chat and took detailed notes. Both TH and KP are experienced qualitative researchers. A third researcher provided technical support at the beginning of each workshop.

The NGT facilitation guide and process were internally tested through four mock sessions with the research team under real conditions (e.g. use of different devices and responding in real time) [29]. The research team developed a risk management procedure for technical difficulties and participant distress. All participants were emailed a study information pack and technology guide and completed an online consent form and participant demographic survey prior to the workshop.

During the workshops, the facilitator presented a short, ten-minute narrated and image-based video summarising the six evidence-based interventions (see Fig. 1). This format was used to increase the engagement for participants, some of whom were expected to have low research literacy [35], and to ensure consistency in the presentation of information across NGT workshops. Following the video, participants individually rated the interventions based on their priority for families in Wyndham on a 5-point scale from ‘very low’ to ‘very high priority’. The group then discussed their perspectives on and experience with the interventions, explained their rationale for ratings, and discussed challenges and enabling factors for using intersectoral services (caregiver participants), or providing or referring families living with adversity to these services in Wyndham (service providers). After the group discussion, participants independently completed a second rating of the interventions, contributed to the final group discussion and the workshop was closed.

### Data analysis

Each NGT resulted in a rated list of interventions (quantitative data). The final ratings for the six interventions from all NGT workshops were combined to form consensus across all groups (i.e. service provider groups and caregiver group together). Consistent with previous research, consensus was reached when interventions were rated as high or very high priority by 75% or more participants {Diamond, 2014 #468}. A 75% proportion level was adopted as the definition of consensus because of the mixed stakeholder groups {Jorm, 2015 #119}. This 75% consensus level is the most commonly used threshold to define consensus in Delphi studies {Diamond, 2014 #468} and was adopted by the unpublished Delphi study informing the current study (Sahle et al.).

No statistical analysis is required to interpret these ratings. Participant demographic information (e.g. mean age, gender, ethnicity) was collated using STATA 16 software.

Each NGT workshop was transcribed verbatim by an external professional company and imported into NVivo Release 1.0 for analysis. Three researchers (TH, SH, LC) employed inductive and deductive content analysis to analyse the qualitative data arising from the workshops. All three researchers have a background in public health and LC is a peer researcher. The first author developed a draft coding frame with deductive themes based on the research questions (i.e. family access enabling factors, family access barriers, service level enabling factors, etc.). Inductive content analysis involved close coding to identify content items emerging from the data, and then cross-referencing between all transcripts to develop common content categories, i.e. provisional inferences drawn from statements and observations 36. The three researchers independently coded two transcripts, and then met to review and discuss the emergent codes to reach consensus on the coding framework. Two researchers (TH and LC) then applied the revised coding framework to the six transcripts.

### Ethics

Before each NGT workshop commenced, participants provided written informed consent to take part in the audio-recorded workshop. Participants provided separate consent for quotations to be used. Ethical approval was granted by The Royal Children’s Hospital Human Ethics Research Committee (HREC #62,129).

## Results

Findings are structured around the six intervention types and the themes and sub themes for enabling factors and barriers to their implementation in Wyndham. Participant quotes are labelled as: caregiver (CG) and service provider (SP).

Three of the six evidence-based intervention types included in this study reached consensus: nurse home visiting programs, parenting programs and community-wide programs. Table 3 displays the proportion of stakeholders who rated each intervention as a high or very high priority for Wyndham as well as the proportion per stakeholder group. Given the small sample size, the group proportions should be interpreted as trends.

**Table 3:** Rated priority of six evidence-based interventions

	*n*	Interventions						
Stakeholder type		Parenting programs	School-based anti-bullying programs	Psychological therapy for children exposed to trauma	Community-wide programs	Nurse home visiting programs	Economic and social programs	Total across interventions
*Service providers*		% high or very high priority (n)						
Health	3	100 (3)	66.7 (2)	33.3 (1)	100 (3)	100 (3)	33.3 (1)	72.2 (2)^
Child and family	3	100 (3)	33.3 (1)	66.7 (2)	33.3 (1)	100 (3)	66.7 (2)	66.7 (2)
Social sector	2	100 (2)	50.00 (1)	100 (2)	100 (2)	100 (2)	100 (2)	91.7 (2) ^
Early education and disability inclusion	7	71.4 (5)	71.4 (5)	85.7 (6)	100 (2)	85.7 (6)	85.7 (6)	83.3 (6) ^
Drug and alcohol	2	100 (2)	50.00 (1)	100 (2)	100 (2)	100 (2)	100 (2)	91.7 (2) ^
*Caregivers*	2	100 (2)	100 (2)	50 (1)	50 (1)	100 (2)	50 (1)	75 (2) ^
Overall	19	89.5* (17)	63.2 (12)	73.7 (14)	84.2* (16)	94.7* (18)	73.7 (14)	79.8 (15) ^

*Consensus reached when interventions were rated as high or very high priority by 75% or moreparticipants

^Rounded to the nearest whole number of participants

### Nurse home visiting

All but one participant endorsed nurse home visiting programs as a high or very high priority (94.7%). During the discussion, both caregiver participants reported finding the nurse home visiting programs they had accessed useful. One caregiver explained this was because nurse home visiting had helped her to learn how to parent her child and resolved other concerns that were impacting her wellbeing:

And home visiting programs should be there because during that first month or some period, you need some help from the nurses and we have no idea how to take care of baby. […] I got some problems during that period, but it was resolved by the nurse. (CG2)

Service providers from across sectors explained that nurse home visiting programs act as a gateway for identification of adversities and coordinating other necessary responses for families:

the nurses that are visiting someone's home, that are going to drive some of those other programs, so they might be there to do some of that early intervention work (SP18 drug and alcohol)

An essential part of this gateway was building trust and relationships with families over time, as one child and family worker explained:

When the nurse visits, she gets the vibe of the house or she gets to know the environment. She might not get a clear picture in the first visit or so, but obviously if she's on her third visit, she would have a better idea to sense the situation, if things are going well or if mum needs an extra support or to help in the best possible way (SP06 child and family)

### Parenting programs

Parenting programs were endorsed as a high or very high priority by 17 out of 19 participants (89.47%). Similarly to nurse home visiting programs, caregiver participants felt parenting programs could address the knowledge gap for new parents and provide support to their transition to parenthood. One health service provider described the potential for parenting programs to socialise parents to the service system and help them to learn fundamental skills which freed up their time spent with allied health to focus on other challenges:

So if they are already being linked in with some of those parenting programs from a younger age, then by the time that they come to us, maybe we're not seeing those issues as a first thing …] the psychologist has time to work on some other things (SP37 health)

While recognising the promise of parenting programs, multiple service providers also described the difficulty of engaging parents living with adversity in these programs:

[Vulnerable communities are] much harder to recruit and continue and also [ensure that they] have the headspace to be able to utilize the information. (SP36 education and disability)

### Community-wide programs

Community-wide programs were endorsed as a high or very high priority by 84.22% of participants (*n* = 16). Consistent with the reasoning for nurse home visiting and parenting programs, caregivers and services providers across sectors felt that community-wide programs acted as a gateway to engage families, for families to find out about available services and supports, and link in with such services. One caregiver explained that: “when you have community-wide programs you could make aware what the community could offer for the mental health services.” (CG1). A health professional emphasized the importance of community as a platform for holistic, multi-disciplinary service provision:

we can't do any of this without community. I think that it's the glue to holding everything together in terms of information sharing and collaboration and being the medium of sharing of different skill sets and different professional lenses. (SP17 health)

However, there were mixed perspectives on community-wide programs. While some service providers saw community-wide programs as a place for families to connect and engage with each other and available supports, caregivers and other service providers felt that these programs were less relevant in Wyndham because the community is already close-knit and mobilised. One caregiver explained that “I also gave a low priority to the community-wide program because we have already some community and we have some events with our community.” (CG2). The other caregiver also felt that community-wide programs might be less useful because they are “almost like a fun event rather than having any real purpose” (CG1).

### Interventions that did not reach consensus

School-based anti-bullying programs, psychological therapy for children exposed to trauma, and economic and social programs did not reach consensus. While some service providers identified that psychological therapies could provide a “fresh air” for families to “leave those challenging things at home and […] spend one-on-one time” (SP15 education and disability), other providers and caregivers felt that these interventions were not a priority because they were not the first course of action and were not readily available due to long waitlists. One caregiver explained that unlike home visiting, the effectiveness of psychological therapy also depended on the level of awareness and engagement of the parent as well as their connection to the professional:

because this [is a] psychological thing, initiation from the self doesn't happen that quickly. It might be very hard for me to realize that I am having some issues […] it really depends on when you get that right therapist. […]. (CG1)

Economic and social supports were not seen as a priority because service providers and caregivers felt that these supports were already provided in Wyndham: “Because there's a lot of help in terms of the Centrelink and […] a lot of stuff with just helping through the economic and social service programs.” (CG1). The school-based anti-bullying program had less endorsement because it did not involve early intervention. In reference to the anti-bullying school program, one service provider said: “I'm a huge advocate for naught to five. […] all of my career there's been a huge emphasis on infant mental health.” (SP 17 health).

### Enabling factors and barriers to the implementation of interventions to support families living with adversity in Wyndham

**Fig. 2:**
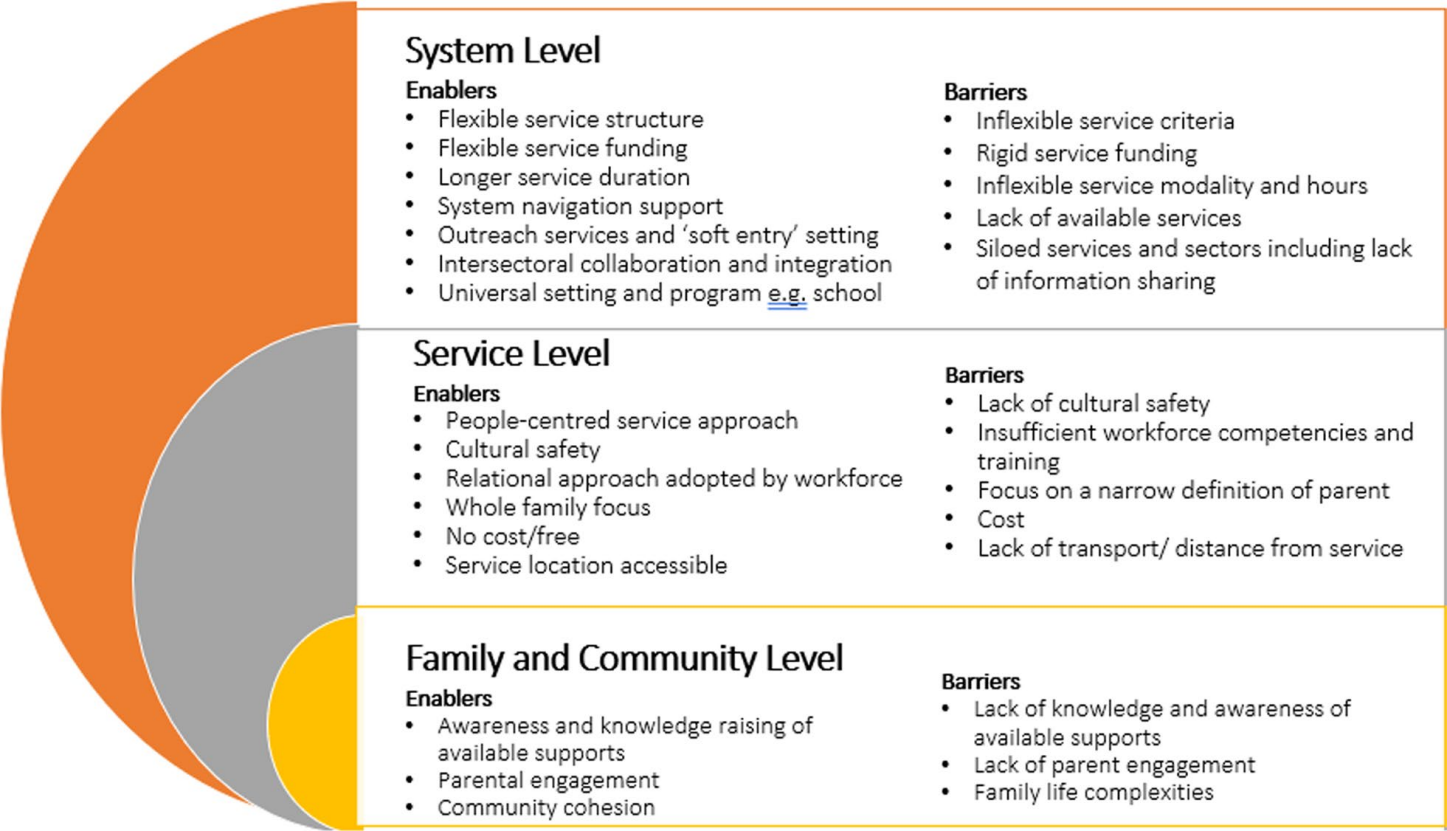
Conceptual model of enablers and barriers to implementation of interventions to support families living with adversity in Wyndham

Several key enabling factors and barriers occurring at the family, service and systems levels were identified for the implementation of interventions to support families. The main themes are summarised in Fig. 2. See Additional file 1: Table A for quotations relevant to each theme.

#### Family and community level

##### Knowledge and awareness of available supports

Caregiver and service providers stated that many families did not know what was available to them:

a part of the challenges that families experiencing, particularly those that come from low incomes, or migrant and with refugee backgrounds, […] don't know what [they]'re entitled to. [they] don't know how to ask for it cause [they] don't know what it is. (SP25 social)

Participants offered suggestions for facilitating knowledge and awareness of available supports and services, including through community groups on social media, word of mouth, through General Practitioners (GPs), maternal and child health nurses, shopping centres and libraries. Multiple participants highlighted the need to provide information in multiple languages given the cultural and linguistic diversity of Wyndham.

##### Parent engagement

Service providers expressed that often the complexity of challenges facing families were an obstacle to their engagement with the agency to uptake services:

…families that are faced with life challenges, they don't always choose the options that are on offer to them. It could be a financial barrier. It could be a mental health barrier. It could be a number of things that are sort of in the way. (SP11 education)

These complexities were highlighted for families with previous negative experiences with the service system (e.g. child protection) and families who were not eligible for Medicare (Australia’s government-funded, free healthcare scheme) or other services due to their temporary visa status. Strategies to promote parent engagement emerged as a key enabler to successful uptake of interventions by all types of services and families, including providing after-hours and outreach service options.

#### Service level

##### People-centred approach to services

A people-centred approach to services was identified as a key enabler to supporting families living with adversity in Wyndham. Specifically, a focus on the needs and preferences of families through providers taking time to build trust and relationships with families. Participants highlighted the need to focus on families’ strengths and assets rather than engaging with families around “*something is broken and we need to fix it*" (SP10 social). Service providers and caregivers also emphasized that services should include the whole family, in particular fathers, as well as grandparents, kinship carers and siblings. Participants positioned this in contrast to the way that many services continued to focus exclusively on mothers. This was seen to be crucial for ensuring cultural safety for Aboriginal and Torres Strait Islander peoples and diverse communities in Wyndham: “[*programs] have to encompass and be mindful of cultural differences*.” (SP9 education).

##### Service funding and modality

Participants identified inflexible service models and service funding models as a key barrier to the provision of services for families living with adversity in Wyndham. Specifically, the provision of services during business hours at a service location. Service providers identified the possilibities of a diverse service offering online service delivery and outreach to better meet the needs of families, albeit recognising the inequities of digital access in Wyndham:

Certainly what I'm seeing now is that for those families that score really highly, they may not feel they can commit to a face to face service because they're anxious, or because of family violence, they can't actually access that service. […] those families with those increased vulnerabilities are requesting home services (SP17 health)

Key barriers to service access for families in Wyndham were related to workforce competencies and supports. Several service providers identified a lack of cultural diversity of the workforce and knowledge about how to work with culturally diverse clients: “… one of the things that's really important to improve on and in our area and in this industry is that cultural knowledge, diversity.” (SP025 social).

Additional access barriers for families included any cost for service and services being located in areas with poor public transport access. One service provider participant providing long-term support for clients described the “*luxury*” of time (SP16 drug and alcohol) which corroborated with other providers acknowledgement that they had increasingly less time allocated with clients over their time working in Wyndham.

#### System level

##### Navigating the system

Support for families to navigate intersectoral services was identified as a key enabler to family access, including comprehensive assessment, care navigation and pathway planning. A caregiver said it would have been helpful if her GP had linked her with all available programs at the beginning of her pregnancy: “Saying, ‘Hey, for the life journey of you and your kid for the next eight years, these are the programs which the gov[ernment] offers." (CG1).

##### Outreach services and ‘soft entry’ setting

Caregivers and most service providers saw outreach services as a gateway to service access, particularly for families with complex life circumstances:

a lot of families that feel completely overwhelmed by parenting in general and the day to day expectations of them as parents, of community members, of families […] more outreach type services would be the answer to that. (SP12 education)

Providers described getting to know families in a safe environment and linking them to a range of supports as the family felt comfortable to share their experiences over time.

##### Intersectoral collaboration

Siloing of services was a key barrier identified by multiple service provider participants. Some providers said they were unaware of all programs available in Wyndham. “*Red tape*” also limited the ability for providers to share client information that would enable clients to seamlessly transition between services. Other providers identified shared network meetings and other mechanisms for information sharing as enablers of collaborative practice: “[it’s] been great to say, ‘What's going on in your space? What's going on in our space?’” (SP13 education).

##### Available services

A lack of available services, particularly allied health, was identified as a key barrier by several service providers: One education service provider explained: “We're supposed to be capacity building educators to support these families in these situations, but we're suggesting things [services] that there's a bottleneck or just an absolute stop. I think that's still a big gap.” (SP13 education).

## Discussion

This study is the first of our knowledge globally to employ a NGT consensus method to prioritise interventions for preventing child mental health problems for children living with adversity from the perspectives of local caregivers and stakeholders. Three interventions reached consensus among the mixed stakeholder groups as being a high or very high priority for implementation in Wyndham: nurse home visiting, parenting programs and community-wide programs. Key rationales were the ability for these interventions to act as a gateway for families to increase their knowledge about topics immediately relevant to them (i.e. parenting), increase their knowledge about available supports and build relationships with service providers.

The study found an alignment between the prioritized interventions and rationales given by caregivers and service providers from a range of sectors. In particular, a focus on relational care approaches that allow for early intervention and prevention of family adversity. An emphasis on relational care and early intervention reflects calls for a focus on the prevention of adversity from research, policy and practice perspectives [37, 38]. Shared goals and common experiences have been shown to facilitate collaboration across health, social, community and education sectors to better meet the needs of families living with adversity [39, 40]. Conversely, a lack of common frameworks and vision between sectors and providers is a well established barrier to effective intersectoral collaboration [41]. Across all interventions, the degree of agreement within participant types ranged from 66.7% for child and family services workers to 92% for social sector and drug and alcohol providers. These differences among members of the same stakeholder group are unsurprising given the broad range of disciplines and backgrounds of staff in professional craft groups, particularly those providing a wide range of child and family interventions. Taken together, these findings underscore the need to establish common frameworks and goals both within and between services as well as with caregivers. Such efforts to foster and strengthen shared understandings between services and families and are crucial for achieving an enabling environment for integrated, people-centred responses to child mental health in Wyndham [23, 42].

Important differences emerged between the priorities of local stakeholders and national level experts in the unpublished Delphi study on which this study was based (Sahle et al.). The qualitative data illuminated some of the factors influencing care prioritisation and decision making at the local level. While community-wide, anti-bullying programs and economic and social interventions were perceived as priorities for Australian children by national experts (unpublished data, Sahle et al.), these interventions are already available in Wyndham and hence seen as a lower priority. The mixed reception about community-based programs from the two caregiver participants indicates the need to cater to multiple preferences and underscores the importance of families being engaged in decisions about their own care needs [43, 44].

The enabling factors and barriers to family service access identified in this study have been previously established, including structural (service availability, wait times, transport, etc.), financial (cost) and cognitive factors (knowledge, awareness of service, etc.) [45, 46]. The study findings underscore the need to adopt a relational approach to service provision with local communities i.e. by building relationships and trust with families over multiple visits. Similarly, cultural competence of the workforce is crucial for responding to needs of Aboriginal and Torres Strait Islander peoples and culturally and linguistically diverse peoples, and barriers are imposed on these groups when services are not culturally safe [47]. The study findings align with increased recognition that service and system level integration and coordination are key to responding to holistic needs and drivers of complexity for families living with adversity [37, 48].

Our study has several limitations. The low numbers of caregiver participants, particularly men and parents with more than one child, means our findings may not generalise to other caregiver groups. Despite recruiting through social media, we were unable to recruit our desired number of caregivers to this study (*n* = 20). We largely attribute these low numbers of caregiver participants to the COVID-19 lockdowns in place at the time of data collection. Wyndham had one of the highest COVID-19 caseloads and associated negative economic impacts of lockdown in Victoria. Caregiver participants may also not represent the experiences of families not in contact with services, without internet access or low English literacy. These different caregiver groups may have different preferences for taking part in services e.g. outside of business hours, in a non-traditional health setting such as a community centre, etc. Nonetheless, the NGT workshops identified key service features related to flexible and people-centred modes of delivery in which the needs and preferences of the caregiver and family are foregrounded; findings that are consistent with accommodating differences in preference and need among caregiver groups. A further limitation is that we restricted ratings to one round based on a predefined set of interventions and may therefore have limited the level of consensus achieved, including on other interventions. However, the interventions were selected based on rigorous evidence, which is important for promoting evidence-based practice to improve population mental health [49].

Despite these limitations, the study illustrates the value of the NGT consensus method for engaging a diverse range of intersectoral service providers and to a certain degree, caregivers. We found the online format to have high acceptability and accessibility for service providers [29, 30]. The study demonstrates that the NGT method can be successfully adapted for an online setting and mobilised to achieve two research objectives, namely, to reach consensus as well as elicit a deep understanding of local feasibility considerations. The innovative application of the NGT method highlights how existing stakeholder engagement methodologies (i.e. focus groups and individual interviews) can be modified to elicit consensus in addition to rich contextual information. The study also demonstrates that the NGT method can be used in place of Delphi methods for understanding local priorities. Specifically, a key design feature of the NGT method is that several rounds of ratings occur in one session which maximises engagement and access to priority setting for busy professionals and community members. This contrasts with the Delphi method which typically involves several rounds of rating through multiple, separate interactions with participants. As such, this method added depth to our overall CRE project by allowing a comparison of national expert consensus with local priorities. The study also has important methodological implications for future consensus work focused on hard-to-reach populations {Bonevski, 2014 #289}.

## Conclusions

This study is the first of our knowledge globally to employ a NGT consensus method to prioritise interventions for preventing child mental health problems for children living with adversity from the perspectives of local caregivers and stakeholders. Local priorities for Wyndham emerged based on interventions that can act as a gateway for families to increase their knowledge about topics immediately relevant to them (i.e. parenting), increase their knowledge about available supports and build relationships with service providers. The study revealed important alignments and differences both between and within stakeholder groups that underscore the need to strengthen shared understandings between services and families for achieving integrated, people-centred responses to child mental health in Wyndham. Despite the lower than expected number of caregiver participants, the study has makes an important methodological contribution to the field of family adversity and stakeholder engagement. Specifically. this study illustrates that the NGT is an effective method for prioritising evidence-based practice interventions in health settings, engaging local stakeholders, and identifying enablers and barriers to implementation. The innovative application of the NGT method in this study highlights how existing stakeholder engagement methodologies can be modified to elicit consensus in addition to rich contextual information. The process and outcomes of this study provide important contextual information and stakeholder engagement that will increase the likelihood of successful implementation of future integrated responses to child mental health in Wyndham.

## Supplementary Information

**Additional file 1**. Enablers and barriers at the system, service and family and community level.

## Data Availability

The quantitative dataset analysed during the current study is available from the corresponding author on reasonable request. Detailed de-identified quotations from the qualitative data set are reported in the body of this article and in Additional file 1: Table A.

## Abbreviations

NGT: Nominal group technique
ACEs: Adverse childhood experiences
CG: Caregiver
MCH: Maternal Child Health
LGA: Local Government area
SP: Service provider
