# Supplementary material for: Prioritising interventions for preventing mental health problems for children experiencing adversity: a modified nominal group technique Australian consensus study
Source: BMC Psychol. 2021 Oct 24;9:165. doi: 10.1186/s40359-021-00652-0 (PMC8542357; doi:10.1186/s40359-021-00652-0)
Supplement: Supplementary file 1 — Additional file 1. Enablers and barriers at the system, service and family and community level. [file 40359_2021_652_MOESM1_ESM.docx]

# Supplementary Table A

**Table A.1 Family and community enablers and barriers**

| **Main theme** | **Sub theme** | **Enabler or barrier** | **Example quotation** |
| --- | --- | --- | --- |
| Awareness and knowledge of available supports | Awareness and knowledge raising of available supports | Enabler | *“… awareness that these programs are there for parents. That will actually help people to actually access the programs more, … when you start your pregnancy journey, from the hospitals or from the care providers, they should actually educate the people like, "There are these programs available for you in the community etc.” (CG1)*  *“So, I guess advertising in some way really getting the word [out] … word of mouth, the doctors, maternal child health nurses. Even at shopping malls, libraries, things like that. Places with families with young children would be appealing on a regular basis, and in multiple languages. Having information out there so that families know, even just websites you can add to stuff. Social media is a really huge way that people are engaging with information these days. And everything's very much internet social media based.” (SP25 Social services)* |
|  | Lack of knowledge and awareness of available supports | Barrier | *“a part of the challenges that families experiencing, particularly those that come from low incomes, or migrant and with refugee backgrounds, […] don't know what [they]'re entitled to. [They] don't know how to ask for it cause [they] don't know what it is.” (SP25 Social services)* |
| Parental engagement | Engaged parents | Enabler | *“… in terms of engaging dads, we do a lot of outreach work, we do a lot of after-hours work, we do lots of home visits, and we found, particularly with Zoom, we've been engaging a lot more dads…” (SP10 Social services)* |
|  | Lack of parent engagement and mistrust | Barrier | *“… the experience I've had trying to encourage parents to participate it's the ones that are probably doing okay, that go along. And the ones that aren't, they're much harder to recruit and continue and also [to] have the headspace to be able to utilize the information. (SP36 Early education and disability inclusion)*  *“for some families too, […] if they've had traumatic experiences with services before, or been involved with Child Protection, there's a real fear that their kids will be taken off them if they say that they're struggling…” (SP10 Social services)* |
|  | Family life complexities | Barrier | *“…families that are faced with life challenges, they don't always choose the options that are on offer to them. It could be a financial barrier. It could be a mental health barrier. It could be a number of things that are sort of in the way.” (SP11 Early education and disability inclusion)* |

**Table A.2 Service enablers and barriers**

| **Main theme** | **Sub theme** | **Enabler or barrier** | **Example quotation** |
| --- | --- | --- | --- |
| People-centered service approach | Relational approach | Enabler | *“I think having really approachable services and working really collaboratively with families to build that trust, it's that trust and engagement. The engagement is so important, and often we don't allow enough time or resources to engage families, and look at the different ways that families want to be engaged appropriately in a culturally safe way, and all of those elements.” (SP10 Social services)* |
|  | Whole family focus | Enabler | *“all of these things are interventions that work to varying degrees, but it's really about how do we hold the whole view of that family, and assist them to navigate that system, and step them up or down to match that dosage as they need it, through that zero to eight years.” (SP10 Social services)*  *“whether it's dad or grandparents, or foster parents, step-families, all that diversity of family, and how you engage them in all of those different levels of the intervention. And also, the cultural diversity, and our First Nations people, […] what works best, and what sorts of different cultural connections and interventions you need to be able to engage them.” (SP10 Social services)* |
|  | Strengths-based and community led service approach | Enabler | *“If you're saying, "Okay, you're doing these parts well, but these are the things we've got to fix," like it's kind of saying, "Well something is broken and we need to fix it," whereas coming from that strength-based approach can have that flow on effect to different areas of a family's life also.” (SP10 Social services)* |
|  | Narrow focus on mothers | Barrier | *“overall, while some individual organizations are doing things differently, there is still this gender imbalance of kinds, where mum is still the focus, and the responsibilities are still heaped on mum, and that could make dads invisible, and not as accessible to services, and things like that also. (SP1 Child and family)* |
| Flexible service funding and modality | Flexible service funding and modality | Enabler | *…with online capacity […] we've been able to engage with families in different ways, have more flexibility engaged with other family members who we might not have been able to before. (SP1 Child and family)*  *“how do we fund and allow those communities to have some ownership over the way in which those services are delivered to their community* *because if we try to impose that on them, they're not going to come.” (SP10 Social services)* |
|  | Inflexible service modality and hours | Barrier | *“What I'm identifying a lot recently is that a lot of parenting programs, […] they're often at times that don't actually meet the needs of community and families.” (SP7 Child and family)* |
| Cultural safety | Culturally safe approach | Enabler | *“As a workforce, are we inclusive in even understanding what culture is? So, I think to deliver something like that, I think it needs to sort of come in at the top level as well so that staff delivering those programs are comfortable and there's no barriers.” (SP17 Child and family)* |
| Workforce competencies and supports | Lack of cultural safety | Barrier | *“And our system hasn't really confronted the racism that’s endemic, and also unconscious, and we don't realise. So […] often people from migrant backgrounds will feel safer with a worker who has a migrant background.” (SP36 Early education and disability inclusion)*  *“… one of the things that's really important to improve on and in our area and in this industry is that cultural knowledge, [and] diversity.” (SP5 Child and family)* |
| Service cost | Free | Enabler | *“… a lot of parenting programs [are] free, which is fabulous…” (SP7 Child and family)* |
|  | Cost for service | Barrier | *“Definitely any costs involved, [is] a huge barrier I would say.” (SP1 Child and family)* |
| Inaccessible service location |  | Barrier | *“Transport and there just not being the means for families to reach community hubs or services in Wyndham is a really big concern and issue.” (SP7 Child and family)* |

**Table A.3 Systems enablers and barriers**

| **Main theme** | **Sub theme** | **Enabler or barrier** | **Example quotation** |
| --- | --- | --- | --- |
| System navigation support |  | Enabler | *“I think it would be good to have a review process, if there's an intake, an assessment, and to have everybody from each program sitting at the table, and then to be able to support which path they want to take at first, or how do they want to go through the program, so do they need all the programs, is there anything that is missing that they would like the support with, but then to avoid to have to tell the same story to all the different programs.” (SP16 Drug and alcohol)*  *“… how do we just find a way to engage a client, and then ensure that we're doing really proper pathway plans for clients, so that they're going to the next service, and that next service is aware of what's been going on for the family, so they don't drop out and get lost.” (SP18 Drug and alcohol)* |
| Outreach services and ‘soft entry’ setting |  | Enabler | *“… families with those increased vulnerabilities are requesting home services and that we can tailor it more to the needs of the family with maybe three different kids of different ages.” (SP17 Child and family)*  *“… we deliver a lot of parenting programs in the area and we have found out one thing would link to the other. […] So, we would maybe get to know that they are in a family, if they have some issues going on with child protection or family violence or something. And then we need to refer them to other services and organizations for more support.” (SP6 Child and family)* |
| Intersectoral collaboration | Mechanisms for information sharing and collaboration | Enabler | *“… hopefully having a hub, where families are engaged […] and then there's services that can wrap around each individual family, and meet each individual family's needs, that's where I hope we can get to. […] I know we're getting there slowly. (SP1 Early education and disability inclusion)*  *“… consistent intake assessment case planning between all the agencies in Wyndham would just be so awesome because the service system is so siloed.” (SP10 Social services)* |
|  | Siloed services and sectors | Barrier | *“And even as services, if we don't know that those programs are available, that we then also have difficulty in linking our clients and families with them. So, access is huge, I think.” (SP25 Social services)* |
|  | Lack of information sharing | Barrier | *“… There's a bit of red tape in there, and the ability to email and things like that. They've got policies, certain policies and things we've talked about over the years. It's not always viable. Sometimes you have to have a conversation about a client. But they can't do that, because we can't talk about the client in the email.” (SP13 Early education and disability inclusion)* |
| Lack of available services |  | Barrier | *“You've got all those people who may be in these situations. But this can escalate things further when they're having to wait to get their child in to see a paediatrician or a psychologist or a speech therapist. There is just astronomical waiting lists for public health [services].” (SP13 Early education and disability inclusion)*  *“… coming from me working with younger families where we've got our own waitlist, our psychologists [have] got their own waitlist.” (SP37 Health)* |
